# The role of connectedness in haptic object perception

**DOI:** 10.1038/srep43868

**Published:** 2017-03-02

**Authors:** Myrthe A. Plaisier, Vonne van Polanen, Astrid M. L. Kappers

**Affiliations:** 1 Department of Human Movement Sciences, Vrije Universiteit Amsterdam, The Netherlands; 2 Motor Control Laboratory, Movement Control and Neuroplasticity Research Group, Biomedical Sciences Group, Department of Kinesiology, KU Leuven, Belgium

## Abstract

We can efficiently detect whether there is a rough object among a set of smooth objects using our sense of touch. We can also quickly determine the number of rough objects in our hand. In this study, we investigated whether the perceptual processing of rough and smooth objects is influenced if these objects are connected. In Experiment 1, participants were asked to identify whether there were exactly two rough target spheres among smooth distractor spheres, while we recorded their response times. The spheres were connected to form pairs: rough spheres were paired together and smooth spheres were paired together (‘within pairs arrangement’), or a rough and a smooth sphere were connected (‘between pairs arrangement’). Participants responded faster when the spheres in a pair were identical. In Experiment 2, we found that the advantage for within pairs arrangements was not driven by feature saliency. Overall our results show that haptic information is processed faster when targets were connected together compared to when targets were connected to distractors.

When we enclose an object in our hand, we can extract information about this object such as its weight, shape and the material it is made out of. This haptic information enables us to recognize objects quickly and accurately without having to look at them^1^,^2^. We can even efficiently extract information about multiple objects simultaneously held in our hand. For instance, the number of objects can be determined quickly and accurately^3^,^4^. Furthermore, when holding a set of objects, we can quickly detect whether a specific object is present among the other objects^5^,^6^. Besides detecting features, an important step in recognizing what we are holding in our hand is associating haptic information with the correct object. This means that all information from an object has to be assigned to this particular object and segmented from information from another object. Very little is known about this process in the case of haptic perception of handheld objects. In the present study, we make a start into gaining insight into this crucial step for haptic object perception.

Search tasks have been used to investigate the processing speed of specific features of objects held in the hand. In a typical haptic search task, the participant is asked to detect a target between varying numbers of distractor items^7^. For instance, the task could be to detect an object with a certain shape^5^ or roughness^7^,^8^,^9^, an object with a deviating temperature^10^, or whether one of the objects is more compliant^6^. It has also been shown that haptic judgment of the number of objects or targets that are held in the hand is fast and accurate for small numerosities^3^,^4^,^11^. Adding distractors impairs haptic numerosity judgment in a similar way as it does in vision^12^,^13^. Haptic enumeration is more difficult for target and distractor combinations in which the target is less salient than the distractor.

The presence of a target object might be determined by detecting the presence of a certain feature. However, to enumerate a set of targets, the target objects need to be separated from the distractor objects. When these targets and distractors are parts of a composed object, separating targets from distractors might be more difficult and enumeration more time consuming. How do we determine whether haptic inputs belong to a single object? We would like to suggest that in haptics a possible cue in this process is movability, because parts of the same object move congruently. The more freedom of movement between parts, the less likely it is that they belong together. A previous study has shown that haptically distinguishing an object from its background (i.e. figure-ground segmentation) is easier when the object can move with respect to the background^14^. That means that movability is a cue for assigning certain inputs to an object and others to the background. Movability therefore plays a role in the segmentation of a haptic scene. Other indications that movability is an important cue for haptic processing of objects stem from haptic search studies. Movability has been shown to be a salient feature that is detected efficiently^15^. A special status of movability detection has been suggested in a recent study that showed that movability is a property for which detection takes place independent of localization^16^. When two items (for instance, two spheres) are connected together, their movements become coupled, and they might be interpreted as a single object. How would this affect perceptual processing of these spheres?

In the current study, we investigated how enumeration performance is affected if we connect target and distractor items. Such a connected pair might potentially become a single haptic object. How would that influence perceptual processing of other features of the object(s)? Here, we investigated in two experiments if and how connecting target and distractor items influences haptic perceptual processing.

## Experiment 1

**Figure 1:**
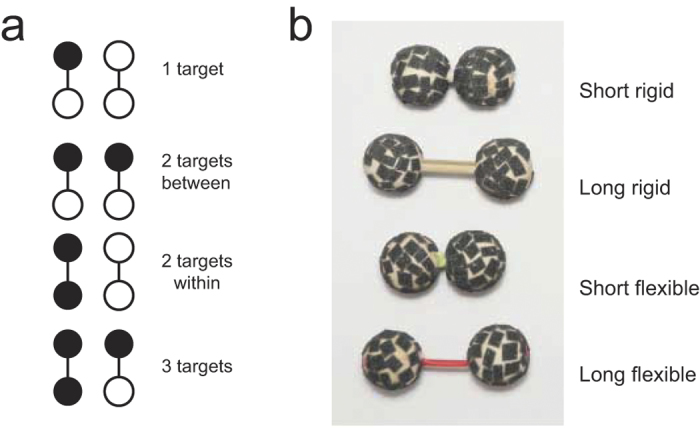
Overview of the stimuli. (**a**) Illustration of the four possible sets of spheres presented. Four spheres were always presented in pairs, and among these there could be one, two or three rough spheres. In the two-rough sphere trials, the rough spheres could be presented within a pair or between pairs. (**b**) The stimuli consisted of spheres that were connected to form pairs. Long and short connectors were used, and these could be either rigid or flexible. The spheres were made out of wood and the spheres depicted here were given a rough surface by sticking patches of sandpaper on them. Smooth spheres had a smooth wooden surface.

Here we introduce a task that we will refer to as a Numerosity Detection Task (NDT). In this paradigm, spheres were presented four at a time to the participant. In each trial, one, two or three of the four spheres would be rough, and the other(s) smooth (see Fig. 1a). The participant had to determine whether or not there were exactly two rough spheres (targets) in the set, while ignoring the smooth spheres (distractors). As an outcome measure we recorded response times (RT). The four spheres were always connected in pairs. If there were two rough spheres present, the spheres could either be connected together as a single pair (‘within pairs arrangement’) or distributed over two pairs (‘between pairs arrangement’) (see Fig. 1a). The parameter of interest was the possible difference in response times between these two types of connections.

We hypothesize three possible outcomes. First, there might be no difference in response time between the within and between pairs arrangements. Although connecting the spheres might make it easier to, for instance, keep track of which item has already been counted, participants might serially examine each sphere individually regardless of how it is connected to another one. In that case, it would not matter how they were connected. The second hypothesis is that the between pairs arrangement is faster than the within pairs arrangement. To enumerate items we need to distinguish between them. This has also been labeled “item individuation”^17^,^18^. Connecting spheres into pairs could impair this individuation process if the two spheres are now interpreted as a single object. This might make it difficult to distinguish the two target spheres when they are connected into a single pair, while they might be separated more easily when they are connected into different pairs. The third hypothesis is that the between pairs arrangement takes longer to process than the within pairs arrangement. Following the opposite reasoning as above, connecting a target to a distractor might actually make it difficult to distinguish the target from the distractor. This may be especially true when the targets are more salient than the distractors. In that case, connecting a target to a distractor might increase response times leading to slower responses for between pairs arrangements than within pairs arrangements. A second reason to expect this outcome is that processing of items within the same perceptual group has been shown to be faster than for items distributed over different groups^19^,^20^. Perceptual grouping might be induced due to similarity. This means that similar items are grouped together. This is a phenomenon first shown in vision, but recently found in haptics as well^21^,^22^. In the case of within pairs arrangements the same types of spheres are connected, which might result in faster response times due to grouping by similarity.

Whether two connected spheres are perceived as one or as two objects might also depend on the type of connector used. For instance, how strongly two spheres are perceived as a single object could depend on their spatial separation. Therefore, we varied the length of the connectors (short, long). Also, all parts of a single rigid object normally move congruently. For instance, all sides of a cube will rotate congruently. Consequently, the amount of coupling of the movement of the two spheres might affect how much they are perceived to be a single object. This is why we also varied the rigidity (rigid, flexible) of the connection (see Fig. 1b). We expect that the perception of a single object is strong weaker when there is more spatial separation and less congruent movements. Therefore, we hypothesize possible effects of sphere arrangement to be smaller for the longer connectors and for flexible connectors.

## Results

**Figure 2:**
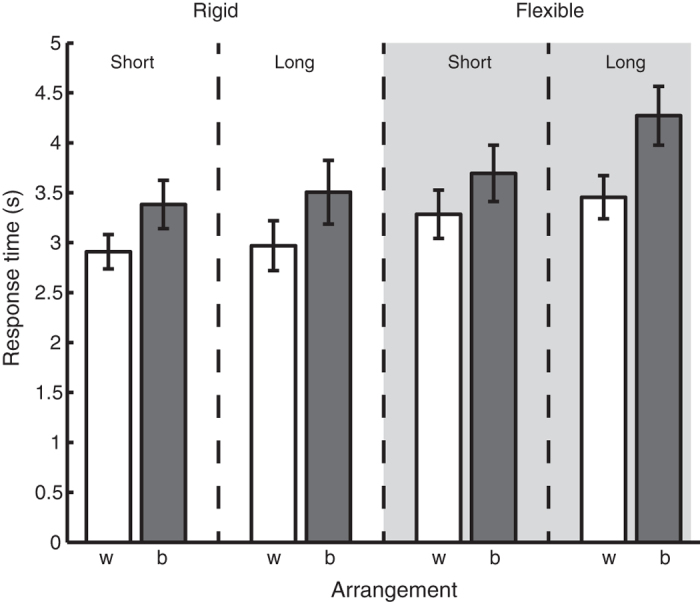
Median response times averaged over participants for the trials in which two rough spheres were present for each of the connector types. RTs are shown for within (‘w’) and between (‘b’) pairs arrangement of the spheres separately. The error bars indicate standard error of the mean. There was a main effect of arrangement and connector type, but no interaction effect.

To investigate the effect of sphere arrangement, we compared the within and between pairs arrangements of the trials in which exactly two rough spheres were present. For each participant the median RT was calculated for each type of sphere arrangement and for each connector type separately. The average over all participants is shown in Fig. 2. It can be seen that the within pairs arrangements yielded smaller RTs than the between pairs arrangements for all connector types. Furthermore, the RTs tended to be longer for the long flexible connector type than for the other connectors. To test whether these effects were significant, a 4 × 2 (connector type × sphere arrangement) repeated measures analysis of variance (ANOVA) was performed on the median RTs. This analysis showed no interaction effect, but did indeed show an effect of connector type (F (3, 24) = 7.9, p = 0.001, η_p_^2^ = 0.50) as well as arrangement (F (1,8) = 5.6, p = 0.003, η_p_^2^ = 0.70). Post-hoc pairwise comparisons with Bonferroni correction showed that RTs for the long flexible connectors were larger than for the short rigid connectors (p = 0.003).

## Discussion

The results were in line with hypothesis three and showed that participants were faster to determine whether there were exactly two rough spheres when they were arranged within a pair than when distributed over two pairs. It is important to keep in mind here that the experiment was designed such that in each trial both pairs had to be examined. If the first two spheres that were detected were both rough (or both smooth), still at least a third sphere had to be examined to know whether the correct response would be ‘yes’ or ‘no’. Therefore, the RT difference between within and between pairs arrangements cannot be caused by differences in the probability of detecting the two rough spheres early in the trial. The only controlled variable was whether two rough spheres were presented within the same pair or divided over two pairs. Consequently, the RT differences reported here were likely due to a perceptual processing difference for the within and between pairs arrangements. The results clearly show performance was faster when the rough spheres were connected into a single pair, supporting the third hypothesis.

Furthermore, there was no interaction between the sphere arrangement and the connector type, indicating that the response time advantage for within pairs arrangements did not depend on the connector type. It is possible that the variation in length of the connectors was not large enough to find an effect. We chose the length of the long connector such that a pair of spheres could still be completely enclosed in the hand. This way short and long connector stimuli could both be explored in the same way, i.e. by enclosing them in the hand. It might be that the length difference of 1 cm was not enough to weaken the effect of the connector. Furthermore, the short flexible connector allowed for less movement between the spheres than the long flexible connector did. Therefore an effect of the rigidity of the connector would be mainly expected for the long flexible connector. This trend is visible in the data.

## Experiment 2

The results of Experiment 1 indicate that counting target spheres was slower when the targets were connected to distractors compared to when targets and distractors were connected to another target or distractor, respectively. We predicted that this may happen because a distractor might be more difficult to ignore when it is grouped with a target. If this is indeed the reason, the effect should be dependent on the saliency of the distractors since more salient distractors are more difficult to ignore. For instance, haptic numerosity judgment has been shown to slow down in the presence of distractors^12^, and this effect depended on the saliency of the targets and distractors.

In this second experiment, we investigated a possible effect of saliency by reversing the target-distractor identity. Rough objects are more salient than smooth objects^7^,^8^. A pair with two rough spheres is therefore more salient than a pair with two smooth spheres. In the case of the between pairs arrangement, each pair consisted of both a smooth and a rough sphere. This means that in that case, both pairs were equally salient. When assessing the number of targets in one pair, the extent to which the participants were distracted by the other pair could depend on the overall saliency of the other pair of spheres. In the within pairs arrangements of Experiment 1 the distractors were smooth, and a pair of smooth spheres should be less distracting than a pair with both a smooth and a rough sphere. Therefore, it is possible that the advantage of the within pair arrangement that was found in Experiment 1 was due to the two target spheres being arranged in a highly salient pair. If so, we expect the RT difference between within and between pairs arrangements to disappear or even reverse if target and distractor identity were reversed. Between pairs arrangement could in this case be faster than within pairs arrangement because a pair of rough spheres is possibly more distracting than a pair consisting of a smooth and a rough sphere. So, if the RT difference between the within and the between pairs arrangement in Experiment 1 was caused by how difficult it was to ignore the one pair while examining the other, we expect this difference to disappear or reverse when the targets are the smooth spheres and the distractors the rough spheres. As we did not find an interaction of type of connector with arrangement, we only used the short rigid connector in this second experiment.

## Results and Discussion

**Figure 3:**
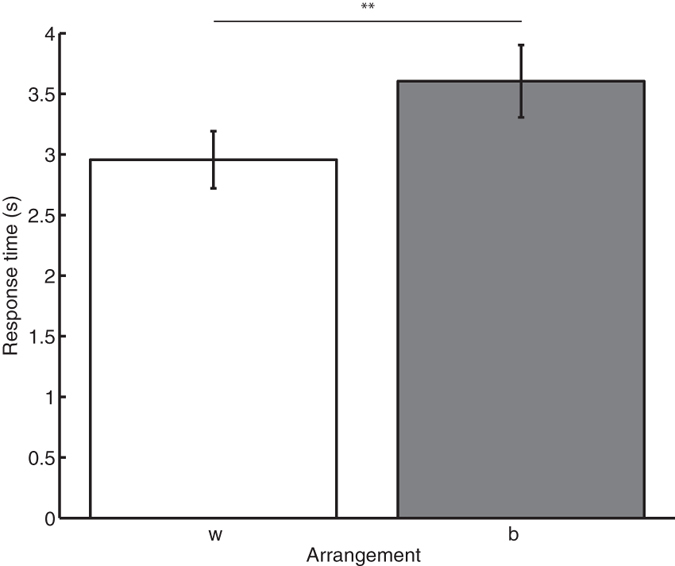
Median response times averaged over participants for the within (‘w’) and between (‘b’) pairs arrangements of the smooth spheres. Spheres were always connected with short rigid connectors. The error bars indicate the standard error of the mean and **indicate p < 0.001.

The RTs averaged over participants in Experiment 2 for both types of arrangements are shown in Fig. 3. Comparing the between and within pairs arrangements, it can be seen that similar to the results in Experiment 1, the RTs for within pairs arrangements were smaller than for between pairs arrangements. A paired samples t-test showed that this difference was statistically significant (t (9) = −4.5, p = 0.0016). This means that the effect of sphere arrangement that was found in Experiment 1 was replicated here in Experiment 2. This shows that the RT advantage of the within pairs arrangement was not due to the high saliency of the target pair compared to the distractor pair. It therefore appears that the RT advantage for the within pairs arrangement was due to the arrangement of the spheres and did not depend on whether the more salient feature defined the targets.

## General Discussion

The results from Experiments 1 and 2 both show that the Numerosity Detection Task (NDT) was performed faster when the target spheres were grouped within pairs than when grouped between pairs. This shows that the number of target spheres was determined faster when the same types of spheres were connected together, even though determining which spheres were connected was irrelevant for solving the task. This might suggest that it was easier to ignore the distractors for within pairs arrangements than for between pairs arrangements. In the NDT, it is important to separate the targets from the distractors, as the number of targets is necessary to perform the task correctly. However, rough (i.e. salient) distractors are usually more difficult to ignore. Given that we find an advantage for within pairs arrangements in both Experiments 1 and 2, it might be that connecting the spheres in a within pairs arrangement facilitated the overall interpretation of the stimulus instead of only making it easier to ignore the distractors. Importantly, in our NDT, explorative movements were necessary for within as well as between pairs arrangements. In both cases, all four spheres had to be examined. Differences in exploration time can therefore not be caused by stimulus properties other than arrangement of the spheres.

No obvious differences in exploratory movements were observed during the experiments for the different types of pair arrangement. As instructed, all participants began by enclosing the stimuli in the hand. After that they generally examined one pair of spheres between the thumb and index finger and used the other fingers to explore the other pair. None of the participants spontaneously reported any differences in strategy used in examining the spheres.

Why would RTs be shorter for the within group arrangements? It has been hypothesized that this would be the case in vision, as suggested by two different types of previous findings^23^. First, visual search seems to be more efficient for items that are within the same perceptual part^19^,^20^. Second, it has been shown that attention can be shifted faster between two locations when these locations are part of the same object^24^. This is referred to as object-guided attention. A notable difference between visual and haptic exploration of a scene or object is that haptics involves quite stereotypical exploratory hand movements. This means that haptic search usually contains a sequential component because the hand or fingers are sequentially moved over certain parts of a scene or object. Comparable to visual search, however, it has also been shown for haptic search that search times are smaller when searching within a perceptual group^25^. Moreover, there is evidence for object-guided attention in touch. It has recently been demonstrated that attentional selection can be object driven, as its time course was shown to be modulated depending on whether the two hands were holding the same object or not^26^,^27^. This suggests that processing times will be reduced when information is contained within the same perceptual group, or object. Therefore, the more the items are interpreted as belonging together, the smaller the response times would be expected to be.

Here we induced grouping by connecting spheres. One could also look at this from the perspective of Gestalt grouping principles. Several recent studies have shown that the Gestalt grouping principles of good continuation, similarity and proximity known from vision also operate in haptic perception^21^,^22^,^25^,^28^,^29^,^30^,^31^. In those studies, however, stimuli consisted of fixed two-dimensional spatial layouts resembling the types of stimuli used in vision. This facilitates comparison between vision and touch. For our study this comparison is not so clear-cut and must be treated with caution. It is, however, useful to draw some parallels to visual grouping principles here. Connecting parts into a single three-dimensional unit or object will consequently mean that the connected parts will stay together and move congruently. This might be considered as the grouping principle known in vision as ‘common fate’. An additional grouping principle that might be at work in our stimuli is grouping by similarity: in the within pairs arrangements the same types of spheres are connected together. Due to the principle of grouping by similarity, the perceptual grouping would be stronger for within pairs arrangements than for between pairs arrangements. This would lead to shorter response times for within pairs arrangements than for between pairs arrangements.

Our results clearly show that information distributed across different groups (or objects) is processed less efficiently than information that is contained within the same object. This study provides a first step gaining insight into the role of connectedness in haptic perceptual processing of 3D handheld objects.

## Methods

### Participants

Ten volunteers (3 male, 1 left-handed, mean age: 23 ± 4 years (SD)) participated in Experiment 1. The data of one of these participants were excluded from the analysis because the instruction was not followed. Another group of 10 participants performed Experiment 2 (3 male, all right-handed, mean age: 22 ± 2 years). All participants were naive as to the purpose of the experiment, and they gave informed consent prior to participating. They received financial compensation for their participation. The experiments were part of a program that was approved by the Ethics Committee of the Human Movement Sciences Faculty at the Vrije Universiteit Amsterdam. The experiments were carried out in accordance with the approved guidelines.

### Stimuli, set-up and design

The stimuli consisted of wooden spheres (1.5 cm diameter). Some of these were made rough by adding a layer of sandpaper (type P120). Sandpaper was applied in small patches distributed over the surface such that the integrity of the overall shape was preserved (the same method was used in ref. 32). We choose to use rough and smooth spheres since this has been shown to be a salient combination^7^,^8^,^32^. In Experiment 1, the spheres were connected to form pairs using either of four types of connectors. They could be connected with a short connector (0.5 cm) or a long connector (1.5 cm). The connector could either be rigid or flexible. The rigid connector consisted of a wooden rod (diameter 3 mm) whereas the flexible connector consisted of a flexible rubber tube (diameter 2 mm). This resulted in a total of four conditions (short rigid, long rigid, short flexible, long flexible) (Fig. 1a). The pairs could either consist of two smooth spheres, two rough spheres or a smooth and a rough sphere (Fig. 1b). The pairs were always presented in sets of two, so there were always four spheres presented at the same time. There could be one, two or three rough spheres in the set. In the case of two rough spheres, these could be grouped within a single pair or distributed between two pairs (Fig. 1). Each trial type (one rough sphere, two rough spheres within, two rough spheres between and three rough spheres) was presented 23 times in each of the conditions, such that the experiment could be performed in two sessions of 1 hour. In Experiment 2, only the short rigid connectors were used, and again each trial type was repeated 23 times.

### Setup and Procedure

**Figure 4:**
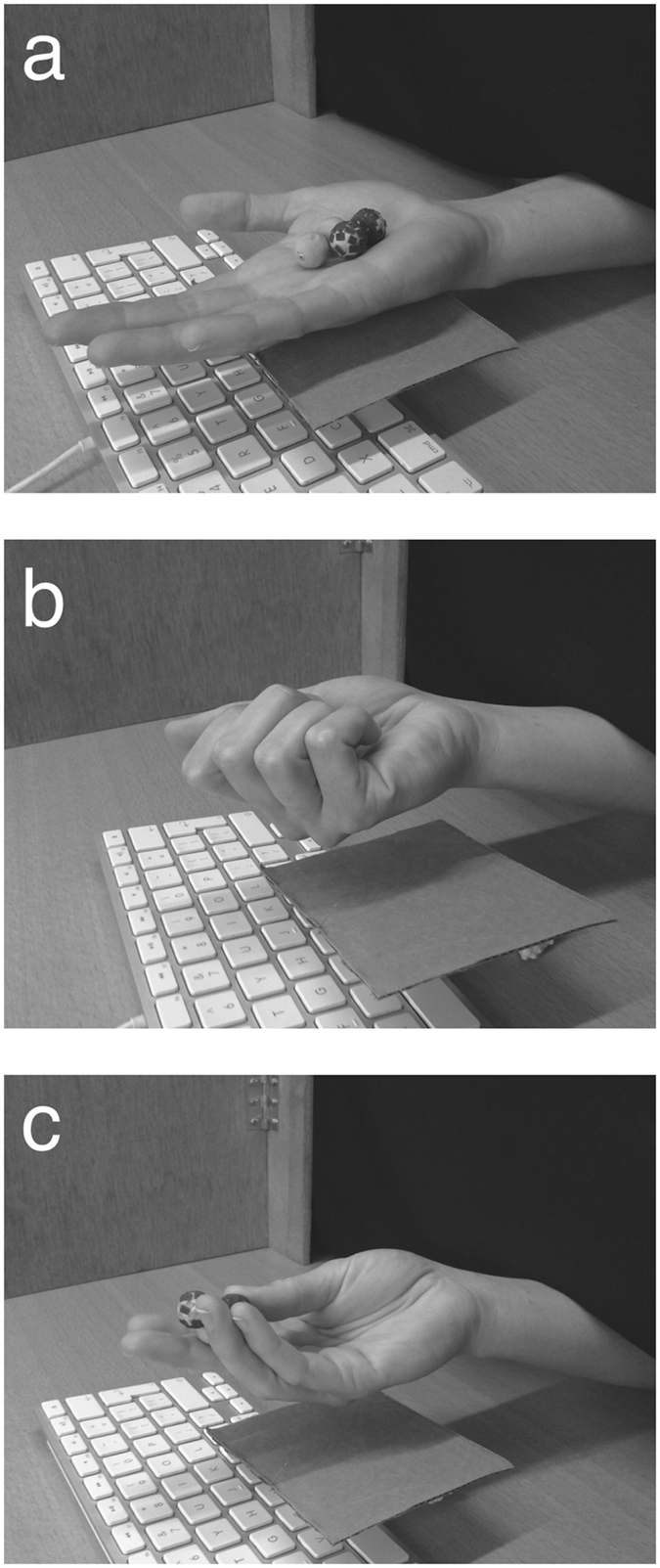
Set-up and procedure for measuring response times. (**a**) The participant placed his or her hand on a platform that was mounted on the space bar of a keyboard. The experimenter placed the stimuli into the hand of the participant. (**b**) The participant lifted and closed their hand. Lifting the hand triggered the onset of the RT measurement. (**c**) Participants were allowed to freely explore the stimuli with the only restriction being that they could not remove items from the hand.

Participants were seated at a table on which a screen with a curtain was placed to prevent the participants from seeing the stimuli or their hand. The setup was placed behind the curtain. The setup consisted of a small platform that was mounted on the space bar of a keyboard. The participants placed their hand on the platform prior to a trial. The release of the space bar triggered the time measurement. Participants placed their dominant hand with the palm facing upwards and a trial was started after the experimenter placed the stimuli in the hand of the participant (Fig. 4a). At that moment, participants moved their hand upward, which started the response time measurement (Fig. 4b). They were free to make any exploratory movements they wanted, but they had to keep the objects in their hand throughout the trial (Fig. 4c). The experimenter ensured that participants complied with this instruction. The measurement was automatically terminated with a vocal response registered with a microphone. This way, RTs were recorded with an accuracy of 21 ± 7 ms (mean ± standard deviation).

In Experiment 1, participants responded with ‘yes’ when there were exactly two **rough** spheres and they responded with ‘no’ when there were one or three **rough** spheres. In Experiment 2, participants responded with ‘yes’ when there were exactly two **smooth** spheres and they responded with ‘no’ when there were one or three **smooth** spheres. In these two experiments, they were told to ignore how the spheres were connected. After giving their response, participants placed the hand back on the platform and the experimenter replaced the stimuli for the next trial. Participants were instructed to respond as quickly as possible, but also to be correct. They received feedback on whether their response was correct after every trial.

Experiment 1 was performed in two sessions on different days. The conditions were blocked, but to minimize order effects half of each condition was performed in the first session and the second half in the second session. The different connector types were presented in different blocks to allow the participant to optimize the exploratory behavior best suited for that specific connector type. To average out possible effects of fatigue or day-to-day fluctuations in performance, all conditions were evenly distributed over both sessions. The order of the blocks was randomized between sessions and across participants. Before each block, a series of ten practice trials was performed to allow the participant to become acquainted with the connector presented in that block. Prior to the first block of the first session a series of 20 practice trials was performed. Experiment 2 was performed in a single block of trials. Prior to the start of Experiment 2, a set of ten practice trials was performed. Incorrect trials were repeated at the end of the block of trials. Because the instruction was that participants should try to be correct, the overall error rates were low (error percentage over all trials averaged over participants: Exp. 1: 2.8% and Exp. 2: 5.6%. In all experiments, only RTs from correctly answered trials were analyzed.

In both Experiments 1 and 2, the trials with one or three rough spheres were included as catch trials only and were not included in the analysis. In the catch trials, participants could sometimes terminate the trial after examining only three of the four spheres. In the trials with two rough spheres, all four spheres always had to be examined to determine the correct answer. As a consequence, catch trials are not informative about the effects of item arrangement.

The effects of connector type and pair arrangement in Experiment 1 were analyzed using a 4 × 2 (connector type × arrangement) Analysis of Variance (ANOVA). We did not use connector length as a separate factor, because connector length is not an independent variable. A longer connector for the rigid type only fixes the two spheres a larger distance apart. As mentioned earlier, the flexible connector only determines the maximum distance between the spheres. The spheres can be pushed closer together with the flexible connector in between. This leads to more degrees of freedom between the two spheres than in the case of a longer rigid connector.

## Additional Information

**How to cite this article**: Plaisier, M. A. *et al*. The role of connectedness in haptic object perception. *Sci. Rep.* **7**, 43868; doi: 10.1038/srep43868 (2017).

**Publisher's note:** Springer Nature remains neutral with regard to jurisdictional claims in published maps and institutional affiliations.
